# Supplementary material for: Younger generations are more interested than older generations in having non-domesticated animals as pets
Source: PLoS One. 2022 Jan 26;17(1):e0262208. doi: 10.1371/journal.pone.0262208 (PMC8791465; doi:10.1371/journal.pone.0262208)
Supplement: S1 File — (DOCX) [file pone.0262208.s001.docx]

**Supporting Information**

**Table S1. Self-reported Genders.**

| **Study** | **Self-reported Gender** | **Frequency** |
| --- | --- | --- |
| Sloth | Female | 918 |
| Sloth | Male | 833 |
| Sloth | Other gender | 3 |
| Sloth | Prefer to self-describe: genderqueer | 1 |
| Sloth | Prefer to self-describe: nebularian | 1 |
| Sloth | Prefer to self-describe: nonbinary | 2 |
| Sloth | Prefer to self-describe: transman | 1 |
| Sloth | Prefer not to say | 8 |
| Python | Female | 873 |
| Python | Male | 892 |
| Python | Other gender | 7 |
| Python | Prefer to self-describe: agender | 1 |
| Python | Prefer to self-describe: gender fluid | 1 |
| Python | Prefer to self-describe: nonbinary | 1 |
| Python | Prefer not to say | 10 |

Table S1 Legend. This table provides participants’ responses to the question “Which of the following best describes your gender?” Response options were “female,” “male,” “other gender,” “prefer to self-describe,” and “prefer not to say.” In the case that respondents selected “prefer to self-describe,” an open text field for self-description was offered. For statistical analysis, genders for analysis were “male,” “female,” and “other” following [75].

**Text S1. Additional Context Evaluation for Sloth.**

An additional visual context was evaluated by survey respondents to inform internal decision-making at Lincoln Park Zoo. At the time of the study design, Lincoln Park Zoo intermittently shared video on YouTube of a resident sloth in his primary zoo habitat feeding from the gloved hand of a member of Lincoln Park Zoo’s animal care staff. Only the gloved hand was shown on video; a full person was never shown. This program, “Lettuce with Luigi,” is referred to as LWL below.

In order to evaluate whether the visual context of LWL inadvertently contributed to interest in sloth ownership by viewers, we included a still image obtained from one video (Fig. S1) in the online survey. Because the image differed in many ways (i.e., image orientation, sloth body orientation, size, posture), we did not include this condition in the analysis reported in the Main Document. However, the results of an ordinal logistic regression model primarily intended for internal evaluation of the LWL program are reported below.

Comparable to the other conditions (Main text Fig. 1), 299 survey respondents viewed this image. As in the model reported in the main text, the dependent variable was four-level ordered categorical variable (the respondent’s Likert response), and the predictor variables were visual context (fixed categorical predictor with *the six levels in Fig 1 and an additional level referred to as “LWL”*), respondent age (fixed categorical predictor binned by generation as follows: Gen Z (age at time of survey 18-24), Millennial (age 25-40), Gen X (age 41-56), Boomers II (57-66), Boomers I (67-75), Post War (76-93)) and respondent gender (fixed categorical predictor: male, female, other).

To determine whether predictors were significant, we again used the anova function in the CAR package to perform Type II likelihood ratio tests on the ordinal logistic regression models. Results are reported in **Table S2** and **Table S3**, and the predicted probabilities of visual contexts including the LWL condition is shown in **Fig. S2**.

Including the LWL condition does not lead to different conclusions; we again find that generation is a significant predictor of interest in sloth ownership whereas gender and visual context are not. The probability of reporting interest in sloth pet ownership after viewing the LWL stimulus is not dramatically different from the other visual contexts (**Fig. S2**).

**Figure S1. Visual Stimulus Extracted from the Lincoln Park Zoo program “Lettuce with Luigi.”** Reprinted from Facebook under a CC BY license, with permission from Lincoln Park Zoo, original copyright 2019.


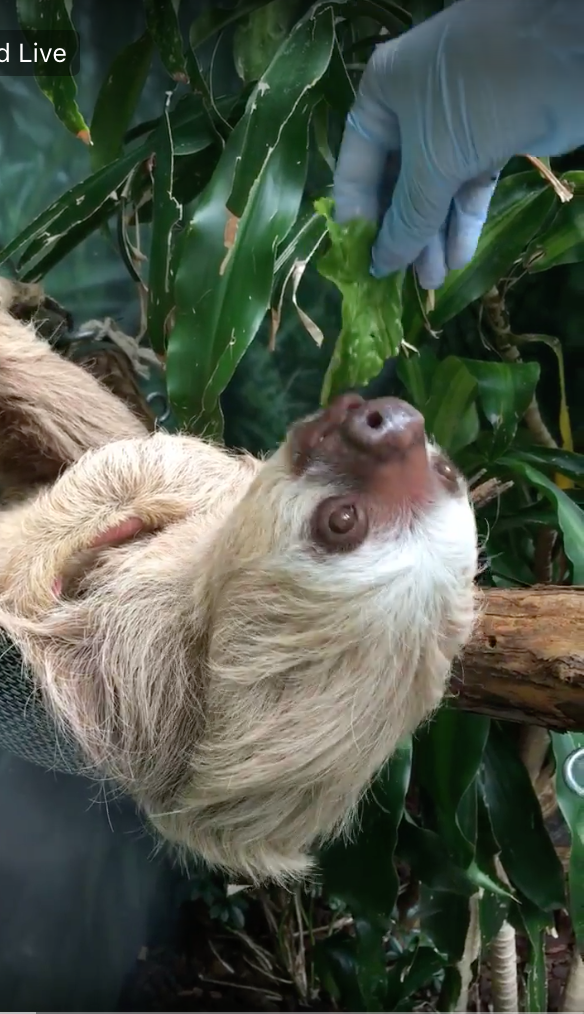


**Table S2. Likelihood Ratio Test Results for Ordinal Logistic Regression Model Including the “LWL” Condition.**

| **Sloth Model including LWL** | ***χ*^2^** | **df** | **P-value** |
| --- | --- | --- | --- |
| Fixed Factors |  |  |  |
| Context | 5.428 | 6 | 0.4902 |
| Gender | 4.889 | 2 | 0.0867 |
| **Generation** | **65.217** | **5** | **1.011 e-12** |

Table S2 Legend. The results of the likelihood ratio test on the data set that includes the LWL condition are similar to the results obtained without the LWL condition (reported in the main text). Again, there is a significant effect of generation, and context and gender are not statistically significant.

**Table S3. Ordinal Logistical Regression Results for the Sloth Experiment Including the “LWL” condition.**

| **Predictor** | **Coefficient** | **Lower-95** | **Upper-95** | **S.E.** | **Odds Ratio** |
| --- | --- | --- | --- | --- | --- |
| Context_Naturalistic Zoo Habitat | -0.0428 | -0.3371 | 0.2515 | 0.1501 | 0.9581 |
| Context_Keeper Contact | -0.1622 | -0.4554 | 0.1308 | 0.1495 | 0.8502 |
| Context_LWL | 0.1269 | -0.1694 | 0.4234 | 0.1512 | 1.1353 |
| Context_Educational Perch | 0.0590 | -0.2353 | 0.3535 | 0.1502 | 1.0609 |
| Context_Educational Perch with Visitor Contact | 0.1052 | -0.1890 | 0.3995 | 0.1501 | 1.1110 |
| Context_Public Setting (Yoga) | -0.0416 | -0.3373 | 0.2540 | 0.1508 | 0.9592 |
| Gender_Male | 0.0287 | -0.1871 | 0.1297 | 0.0808 | 0.9717 |
| Gender_Other | -0.9807 | -1.8789 | -0.1097 | 0.4468 | 0.3751 |
| Generation_ Millennial | 0.0912 | -0.1949 | 0.3775 | 0.1460 | 1.0955 |
| Generation_ Gen X | -0.3398 | -0.6502 | -0.0295 | 0.1583 | 0.7119 |
| Generation_Boomers II | -0.7810 | -1.1604 | -0.4032 | 0.1931 | 0.4580 |
| Generation_Boomers I | -1.0471 | -1.5905 | -0.5123 | 0.2746 | 0.3510 |
| Generation_Post War | -1.5160 | -3.1325 | -0.0985 | 0.7483 | 0.2196 |

Table S3 Legend. The reference value for visual context was the control condition, the reference value for gender was female, and the reference value for generation was Gen Z. Original coefficients are scaled in terms of logs and we provide the exponentiated odds ratios as well.

**Figure S2. Predictor Effect Plot Showing the Role of Visual Context on Interest in Sloth Ownership.** Predictor effect plots provide graphical summaries for fitted regression models by averaging and conditioning the other predictor variables to summarize the role of a selected focal predictor in a fitted regression model [64].

**
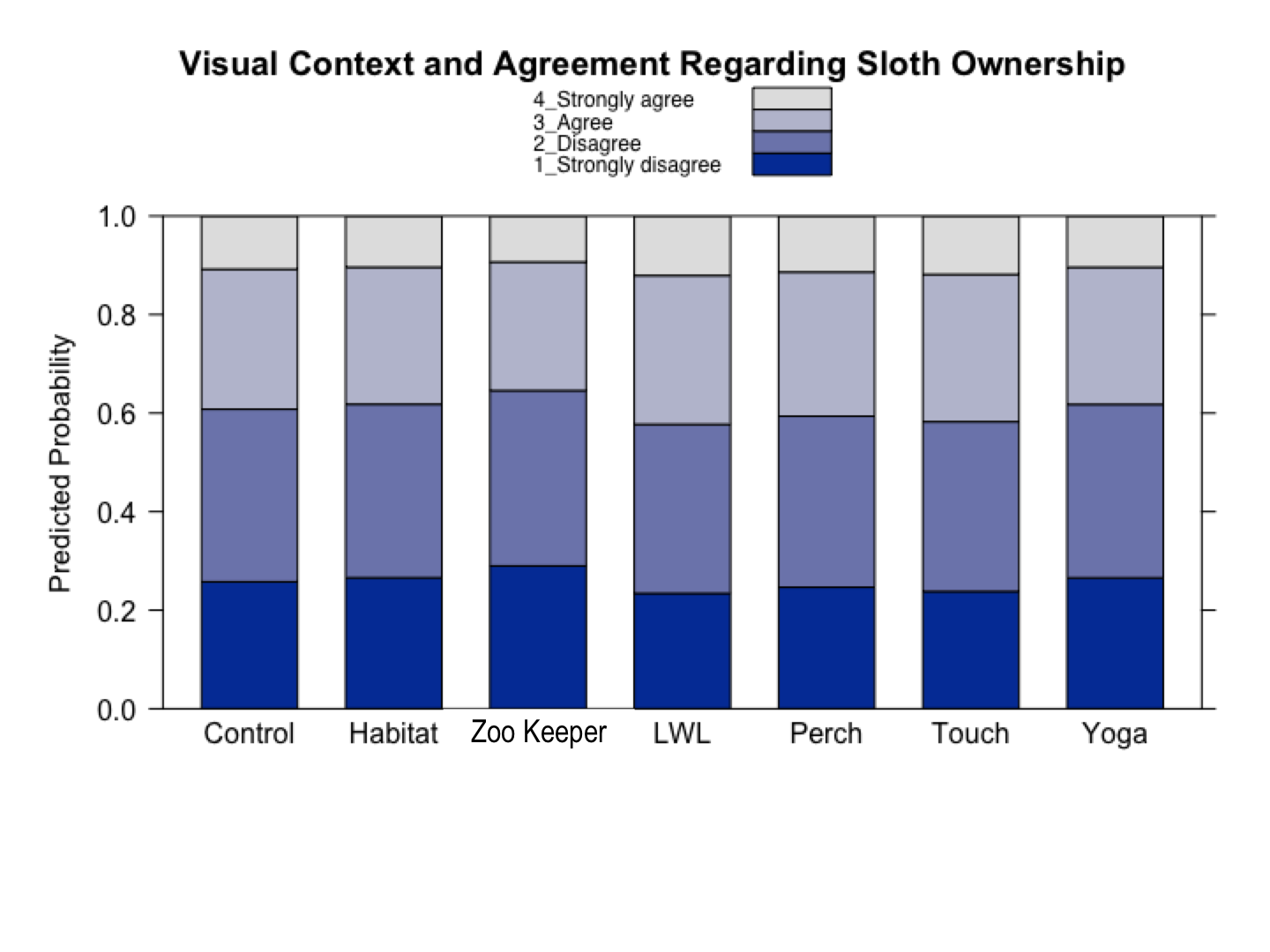
**
