# Supplementary material for: Younger generations are more interested than older generations in having non-domesticated animals as pets
Source: PLoS One. 2022 Jan 26;17(1):e0262208. doi: 10.1371/journal.pone.0262208 (PMC8791465; doi:10.1371/journal.pone.0262208)
Supplement: Attachment — Submitted filename: PONE-D-21-28115_reviewer.pdf [file pone.0262208.s005.pdf]

PLOS ONE

Younger generations show persistent interest in non-domesticated animals as pets

--Manuscript Draft--

**Manuscript Number:**	PONE-D-21-28115
**Article Type:**	Research Article
**Full Title:**	Younger generations show persistent interest in non-domesticated animals as pets
**Short Title:**	Generational differences in non-domesticated pet ownership
**Corresponding Author:**	Katherine A. Cronin<br><br>UNITED STATES
**Keywords:**	animal welfare, public perception, human-animal relationships, zoos, tourism
**Abstract:**	The trade and private ownership of non-domesticated animals has detrimental effects on individual animals and their wild populations. Therefore, there is a need to understand the conditions that motivate and dissuade interest in non-domesticated pet ownership. Past research has demonstrated that the way in which non-domesticated animals are portrayed in images influences the public's perception that they are suitable as pets. We conducted an online survey of people residing in the United States to investigate how images that could be realistically captured in the zoo and broader tourism industries impact the degree to which people viewing the images report interest in having that animal as a pet. We focused on two species, reticulated pythons (Malayopython reticulatus) and two-toed sloths (Choloepus hoffmanni), and presented each species in six different visual contexts. After viewing an image, respondents reported interest in pet ownership on a four-point Likert scale. Each species was studied separately in a between-subjects design and results were analyzed using ordinal logistic regression models. Thirty-nine percent of respondents reported interest in sloth pet ownership, and 21% reported interest in python pet ownership. However, contrary to our hypotheses, we found that viewing these species in different visual contexts did not significantly affect survey respondents' reported interest in having either species as a pet. Male respondents reported more interest in python pet ownership, whereas there were no significant differences between genders regarding interest in sloth ownership. Finally, generation was a significant predictor of interest in both sloth and python pet ownership, with younger generations reporting more interest in having these species as pets. We consider how modern media exposure to animals in unnatural contexts may relate to the generational effect that we report and discuss priorities for future research to better understand the development of individual interests in non-domesticated pet ownership.
**Order of Authors:**	Katherine A. Cronin
	Maureen Leahy
	Stephen R. Ross
	Mandi Wilder Schook
	Gina M. Ferrie
	Andrew C. Alba
**Additional Information:**	
**Question**	**Response**
**Financial Disclosure**<br><br>Enter a financial disclosure statement that describes the sources of funding for the work included in this submission. Review the submission guidelines for detailed requirements. View published research	The authors received no specific funding for this work.

Powered by Editorial Manager® and ProduXion Manager® from Aries Systems Corporation

articles from *PLOS ONE* for specific examples.<br><br>This statement is required for submission and **will appear in the published article** if the submission is accepted. Please make sure it is accurate.<br><br>**Unfunded studies**<br>Enter: *The author(s) received no specific funding for this work.*<br><br>**Funded studies**<br>Enter a statement with the following details:<br>• Initials of the authors who received each award<br>• Grant numbers awarded to each author<br>• The full name of each funder<br>• URL of each funder website<br>• Did the sponsors or funders play any role in the study design, data collection and analysis, decision to publish, or preparation of the manuscript?<br>• **NO** - Include this sentence at the end of your statement: *The funders had no role in study design, data collection and analysis, decision to publish, or preparation of the manuscript.*<br>• **YES** - Specify the role(s) played.<br><br>* typeset	
**Competing Interests**<br><br>Use the instructions below to enter a competing interest statement for this submission. On behalf of all authors, disclose any competing interests that could be perceived to bias this work—acknowledging all financial support and any other relevant financial or non-financial competing interests.<br><br>This statement is required for submission and **will appear in the published article** if the submission is accepted. Please make sure it is accurate and that any funding sources listed in your Funding Information later in the submission form are also declared in your Financial Disclosure statement.	The authors have declared that no competing interests exist.

Powered by Editorial Manager® and ProduXion Manager® from Aries Systems Corporation

View published research articles from *PLOS ONE* for specific examples.<br><br>**NO authors have competing interests**<br><br>Enter: *The authors have declared that no competing interests exist.*<br><br>**Authors with competing interests**<br><br>Enter competing interest details beginning with this statement:<br><br>*I have read the journal's policy and the authors of this manuscript have the following competing interests: [insert competing interests here]*<br><br>* typeset	
**Ethics Statement**<br><br>Enter an ethics statement for this submission. This statement is required if the study involved:<br><br>• Human participants<br>• Human specimens or tissue<br>• Vertebrate animals or cephalopods<br>• Vertebrate embryos or tissues<br>• Field research<br><br>Write "N/A" if the submission does not require an ethics statement.<br><br>General guidance is provided below. Consult the submission guidelines for detailed instructions. **Make sure that all information entered here is included in the Methods section of the manuscript.**	This study was deemed exempt by the Lincoln Park Zoo Institutional Review Board (IRB-20-01-EX), approved by the Lincoln Park Zoo Research Committee (Project 2020-010), and approved by Disney's Animal Care and Welfare Committee (IR2004) and Scientific Review Committee. Subjects indicated consent to participate online prior to completing the survey, and all data were analyzed anonymously.

Powered by Editorial Manager® and ProduXion Manager® from Aries Systems Corporation

**Format for specific study types**<br><br>**Human Subject Research (involving human participants and/or tissue)**<br>• Give the name of the institutional review board or ethics committee that approved the study<br>• Include the approval number and/or a statement indicating approval of this research<br>• Indicate the form of consent obtained (written/oral) or the reason that consent was not obtained (e.g. the data were analyzed anonymously)<br><br>**Animal Research (involving vertebrate animals, embryos or tissues)**<br>• Provide the name of the Institutional Animal Care and Use Committee (IACUC) or other relevant ethics board that reviewed the study protocol, and indicate whether they approved this research or granted a formal waiver of ethical approval<br>• Include an approval number if one was obtained<br>• If the study involved *non-human primates*, add *additional details* about animal welfare and steps taken to ameliorate suffering<br>• If anesthesia, euthanasia, or any kind of animal sacrifice is part of the study, include briefly which substances and/or methods were applied<br><br>**Field Research**<br><br>Include the following details if this study involves the collection of plant, animal, or other materials from a natural setting:<br>• Field permit number<br>• Name of the institution or relevant body that granted permission	
**Data Availability**<br><br>Authors are required to make all data underlying the findings described fully available, without restriction, and from the time of publication. PLOS allows rare exceptions to address legal and ethical concerns. See the PLOS Data Policy and FAQ for detailed information.	Yes - all data are fully available without restriction

A Data Availability Statement describing where the data can be found is required at submission. Your answers to this question constitute the Data Availability Statement and **will be published in the article**, if accepted.<br><br>**Important:** Stating 'data available on request from the author' is not sufficient. If your data are only available upon request, select 'No' for the first question and explain your exceptional situation in the text box.<br><br>Do the authors confirm that all data underlying the findings described in their manuscript are fully available without restriction?	
**Describe where the data may be found in full sentences. If you are copying our sample text, replace any instances of XXX with the appropriate details.**<br><br>• If the data are **held or will be held in a public repository**, include URLs, accession numbers or DOIs. If this information will only be available after acceptance, indicate this by ticking the box below. For example: *All XXX files are available from the XXX database (accession number(s) XXX, XXX.).*<br>• If the data are all contained **within the manuscript and/or Supporting Information files**, enter the following: *All relevant data are within the manuscript and its Supporting Information files.*<br>• If neither of these applies but you are able to provide **details of access elsewhere**, with or without limitations, please do so. For example:<br><br>*Data cannot be shared publicly because of [XXX]. Data are available from the XXX Institutional Data Access / Ethics Committee (contact via XXX) for researchers who meet the criteria for access to confidential data.*<br><br>*The data underlying the results presented in the study are available from (include the name of the third party*	All relevant data are within the manuscript and its Supporting Information files.

and contact information or URL).<br>• This text is appropriate if the data are owned by a third party and authors do not have permission to share the data.<br><br>* typeset	
Additional data availability information:	

Powered by Editorial Manager® and ProduXion Manager® from Aries Systems Corporation

Click here to access/download;Manuscript;Portrayals MS_30Aug21.docx

Younger generations show persistent interest in non-domesticated animals as pets

Katherine A. Cronin[1]*, Maureen Leahy[1], Stephen R. Ross[2], Mandi Wilder Schook[3], Gina M. Ferrie[3], Andrew C. Alba[3]

[1] Animal Welfare Science Program, Lincoln Park Zoo, Chicago, Illinois, United States of America

[2] Lester E. Fisher Center for the Study and Conservation of Apes, Lincoln Park Zoo, Chicago, Illinois, United States of America

[3] Animals, Science and Environment, Disney's Animal Kingdom®, Lake Buena Vista, Florida, United States of America

* Corresponding author
E-mail: kcronin@lpzoo.org

Abstract

The trade and private ownership of non-domesticated animals has detrimental effects on individual animals and their wild populations. Therefore, there is a need to understand the conditions that motivate and dissuade interest in non-domesticated pet ownership. Past research has demonstrated that the way in which non-domesticated animals are portrayed in images influences the public's perception that they are suitable as pets. We conducted an online survey of people residing in the United States to investigate how images that could be realistically captured in the zoo and broader tourism industries impact the degree to which people viewing the images report interest in having that animal as a pet. We focused on two species, reticulated pythons (*Malayopython reticulatus*) and two-toed sloths (*Choloepus hoffmanni*), and presented each species in six different visual contexts. After viewing an image, respondents reported interest in pet ownership on a four-point Likert scale. Each species was studied separately in a between-subjects design and results were analyzed using ordinal logistic regression models. Thirty-nine percent of respondents reported interest in sloth pet ownership, and 21% reported interest in python pet ownership. However, contrary to our hypotheses, we found that viewing these species in different visual contexts did not significantly affect survey respondents' reported interest in having either species as a pet. Male respondents reported more interest in python pet ownership, whereas there were no significant differences between genders regarding interest in sloth ownership. Finally, generation was a significant predictor of interest in both sloth and python pet ownership, with younger generations reporting more interest in having these species as pets. We consider how modern media exposure to animals in unnatural contexts may relate to the generational effect that ~~we report~~ and discuss priorities for future research to better understand the development of individual interests in non-domesticated pet ownership.

Introduction

The trade and private ownership of non-domesticated animals has detrimental effects on individual animals and their wild populations. Unfortunately, non-domesticated (or "non-traditional," or "exotic") pet ownership is widespread and the global market for non-domesticated pets, both legally-traded and illegally-trafficked, is increasing and negatively impacting the status of wild populations of birds, reptiles, amphibians, and mammals [1-3]. A considerable proportion of non-domesticated pets are sourced from wild populations, threatening biodiversity and conservation efforts [1, 2, 4-8].

The welfare of individual animals who are part of this trade and trafficking is often negatively impacted as well. Welfare, or the quality of life as experienced by the animal, is threatened when animals are captured from the wild, both during transport into captivity, and while they are in private homes or poorly regulated business ventures (e.g., animal cafes or touristic photo opportunities). These environments typically introduce behavioral restriction and generally do not meet the animals' needs [e.g., 3, 9-13].

Furthermore, legal or illegal ownership of non-domesticated animals poses risks of disease transmission that are harmful both to humans and nonhuman animals. Diseases are transmitted between animals in crowded conditions during capture, transport and sale, and from animals to humans during close contact [14-20]. Viruses that spread from animal to human hosts have been responsible for massive outbreaks of disease in humans, most recently demonstrated in the global COVID-19 pandemic [21, 22]. With known deleterious effects of non-domesticated animal trade and trafficking on conservation efforts, animal welfare, and human and non-human animal

health, there is a pressing need to understand the conditions that motivate and dissuade interest in non-domesticated animals as pets.

People have become more, rather than less, interested in non-domesticated pets in recent decades [3, 23], and those purchasing these animals are often unaware that they may be contributing to illegal or harmful trade [6]. A recent experimental survey found that people were more likely to be dissuaded from pet ownership due to threats of zoonotic disease and legal ramifications than due to concerns about loss of biodiversity or compromised animal welfare [24]. The increase in non-domesticated pet interest may be due to the widespread availability of the internet and social media, both because of the increased market access for those trying to reach consumers and because of the exposure that people have to images and videos of animals [25-31]. Further, public interest in non-domesticated pet ownership is known to be responsive to media portrayals of animals on Facebook, YouTube, and in popular movies and television shows [32-39[illegible]

Experimental research has also demonstrated that the way in which non-domesticated animals are portrayed influences the public's perception that they are suitable as pets. For example, undergraduate students shown videos of chimpanzees playing an entertaining role in commercials were more likely to answer positively on questions gauging the suitability of chimpanzees as pets than were students who saw videos of chimpanzees in a national park or as part of a conservation commercial [40]. Likewise, people shown images of chimpanzees with a person standing nearby were over 30% more likely to report chimpanzees appealing as a pet compared to people viewing an image of the same chimpanzee without a person nearby [41]. Another investigation that focused on three different primate species (two monkeys and one

prosimian) revealed similar results and demonstrated that when primates were shown in an unnatural setting (an office workplace), people were more likely to consider them suitable pets [42]. Respondents were less likely to judge the animals to be suitable pets if there was no human present, or if the primate was shown in a naturalistic, forested setting. Similar results were also found in a recent study surveying interest in pet ownership among visitors to a combination zoo-amusement park. In this study, visitors were shown images of several different species superimposed in five varying contexts, and interest in pet ownership was lowest in the only condition that did not have a human present in the image [29]. The authors also considered whether subtle differences in the attire of the person in the photo influenced perception, but found no significant effects of attire on perceptions about pet ownership. Taken together, these studies indicate that the environment or context in which non-domesticated animals are presented, and the presence of humans nearby or in contact, can affect the perception that non-domesticated animals are suitable pets. Some organizations have responded to this research by dissuading the sharing of images with characteristics known to encourage the perception of non-domesticated animals as pets [43-45].

The question of how to responsibly portray non-domesticated animals in a way that does not inadvertently encourage pet ownership is especially relevant for zoos where millions of people are regularly brought into close proximity with a diversity of animal species. Zoos adopt many strategies to connect people with wildlife. Many zoos offer visitors the opportunity to view animals in naturalistic zoo exhibits. Additionally, many zoos facilitate up-close experiences with animals, and these experiences may or may not involve physical contact between visitors and the animals [46]. If and how these different experiences foster compassion for animals and promote

pro-conservation behavioral changes in visitors is a topic of current interest [47-49]. These various opportunities offered to zoo visitors generate visuals, both real time and in the form of photographs and videos shared personally or widely on the internet, which may inadvertently influence interest in pet ownership. Although many zoo professionals provide responsible interpretation and conservation messaging during these live opportunities, sometimes even directly advising against non-domesticated pet ownership, this messaging can be lost if zoo visitors walk by without listening to message points, or the public views images on the internet later without context.

Here, we focus research efforts on these portrayal effects in relation to reticulated pythons (*Malayopython reticulatus*) and two-toed sloths (*Choloepus hoffmanni*). These two species are popular in zoos and commonly used in up-close experiences with visitors [46], their demand in the pet trade is negatively impacting wild populations [50, 51], and individuals of both species are likely to experience poor welfare in the private pet trade [51, 52]. While the study was not designed to test for differences between the two species, we were interested in studying two species that differ in their attributes in a way that may modify people's attitudes toward them, including their perceived cuteness and vulnerability, and their physical similarity to humans [53]. The goal of the present study was to establish the prevalence of interest in pet ownership for these two species, and to question whether visual images that are commonly witnessed or photographed in zoo or tourist settings impact people's interest in having that animal as a pet. To do so, we presented online survey respondents with one of several different images showing the animals in varying environments and contexts. Based on past research, we hypothesized that people would report more interest in pet ownership after viewing images of animals in non-

naturalistic environments and images of animals with humans in the image. Furthermore, given previous research showing effects of gender and age on animal attitudes [29, 54-57], we also considered how gender and age impacted pet interest.

Materials and methods

Stimuli

Twelve visual stimuli were created for this experiment (Fig. 1). One identical animal image for each species (python and sloth) was used to create all stimuli for that species. Different backgrounds were applied to portray each species in the natural and unnatural contexts of interest, and a simple green background was applied to create control stimuli for each species. Stimuli materials were gathered from open-source photos or taken by the authors. Stimuli were created using Adobe Photoshop 2020 version 21.2.

Survey Design

Surveys were created using Qualtrics software, Version January 2021 (Qualtrics, Provo, UT, www.qualtrics.com) and administered online using Amazon's Mechanical Turk (MTurk) (www.mturk.com), a website for conducting behavioral research that provides access to a large and diverse subject pool that pays participants [58]. Potential participants were recruited from MTurk's worker base. Workers resided in the United States and searched for our survey using keywords "survey," "attitude," "opinion," and/or "perception," read a brief description of the survey, and were notified of the compensation provided for completing the survey ($0.50),

before agreeing to take the survey. Surveys were administered in a between-subjects design such that each respondent only saw one stimulus type.

The survey began with the presentation of one randomly selected stimulus (Fig. 1) for 15 sec. Respondents could not advance to the survey statement until 15 sec had elapsed. There was no image presented concurrent with the survey statement. The statement they were shown was either "I would like to have a sloth as a pet," or "I would like to have a python as a pet," depending on which treatment to which they were randomly assigned. Responses were provided via a 4-point Likert scale with the terms "strongly disagree, disagree, agree, and strongly agree" as response choices. After the respondent made their selection, they were presented with an additional 23 questions that were the focus of a separate study and are not interpreted here. Finally, respondents entered demographic information including gender and age. There was no time limit for individual questions, and respondents had a maximum time of 20 minutes to complete the entire survey. Respondents were compensated if they completed the survey in full. Survey data collection occurred from Jan 11 – 13, 2021.

No animals were removed from their primary habitat nor physically handled for the purposes of this study. This study was deemed exempt by the Lincoln Park Zoo Institutional Review Board (IRB-20-01-EX), approved by the Lincoln Park Zoo Research Committee, and approved by Disney's Animal Care and Welfare Committee (IR2004) and Scientific Review Committee.

Data Analysis

Survey responses were downloaded from Qualtrics, organized in Microsoft Excel, and analyzed in R version 3.6.3 [59]. Data were cleaned to exclude subjects who reported their age to be < 18 years (3 respondents), those who provided the same answer to all survey items (34 respondents), and those who completed the full survey in an unrealistic time frame (less than one minute; an additional 10 respondents). The resulting data set included 1785 respondents exposed to a python image and 1767 respondents exposed to a sloth image (see Fig. 1 for details of sample size per condition).

Data were analyzed separately by species due to their different propensity to engender pet interest [53] and due to the impossibility of creating realistic stimuli that differed only by species. Survey responses were analyzed by ordinal logistic regression to estimate the effects of visual context, subject age and gender on interest in exotic pet ownership using the polr function in the R package MASS [60]. For both species, the dependent variable was four-level ordered categorical variable (the respondent's Likert response), and the predictor variables were visual context (fixed categorical predictor with six levels, Fig. 1), respondent age (fixed categorical predictor binned by generation following [61] as follows: Gen Z (age at time of survey 18-24), Millennial (age 25-40), Gen X (age 41-56), Boomers II (57-66), Boomers I (67-75), Post War (76-93)) and respondent gender (fixed categorical predictor: male, female, other). Although additional gender identification was provided by respondents (Supporting Information), these three categories were used for analyses following Lindqvist et al. ~~2020~~. To determine whether predictors were significant, we used the anova function in the CAR package to perform Type II likelihood ratio tests on the ordinal logistic regression models. Assumptions were met for both the python and sloth models; there was no evidence of multi-collinearity and the Brant test [62]

indicated that the parallel regression assumption held for both datasets. The Brant tests were run using the function “brant” in the R package BRANT. Data were visualized using the R package effects [63, 64].

Results and discussion

Overall, 38.71% of respondents indicated agreement that they would like to have a sloth as a pet, and 20.96% of respondents indicated agreement that they would like to have a python as a pet (considering “agree” and “strongly agree” combined, Table 1). The results of likelihood ratio tests to identify whether model predictors were significant are reported in Table 2, and full model results are reported in Tables 3 and 4. Contrary to our hypothesis, the visual context in which the animal was presented was not a significant predictor of interest in pet ownership for sloths nor for pythons (Table 2). Considering sloths, generation was a significant predictor of interest in pet ownership but gender was not (Table 2, 3, Fig. 2). Younger generations tended to agree more with the statement that they would like to have a sloth as a pet. Considering pythons, generation and gender were both significant predictors of interest in pet ownership (Table 2, 4, Fig. 3). Younger generations tended to agree more with the statement that they would like to have a python as a pet, and females reported less interest in python pet ownership than other genders (20.2% of females reported agreement or strong agreement compared to 32.5% of males and 66.7% of respondents of other genders).

This study reveals interest in non-domesticated pet ownership in the United States general population. Pythons were indicated to be pets of interest for 1 out of 5 survey respondents, and sloths for 2 out of 5 survey respondents. This is comparable to past studies that have surveyed

pet interest in response to visual stimuli. In the only previous survey of the general public, Ross et al. [41] reported between 27-37% of respondents reporting interest in chimpanzee pet ownership across their treatment conditions. Leighty ~~and colleagues~~ [42] surveyed zoo visitors on zoo grounds and reported 16-21% of guests were interested in ownership of primate species. Finally, in a survey of visitors to a combination zoo-amusement park, Spooner & Stride [29] report 34-41% of respondents were interested in ownership of non-domesticated species. Of course, agreement with statements about animals making appropriate pets, or statements about being interested in pet ownership, does not equate one-to-one with actual pet ownership. Regardless, given the negative impact of non-domesticated pet ownership on animal welfare and conservation discussed above, these numbers highlight the need to continue to study how to influence the perception that non-domesticated species are interesting pets.

To that end, we attempted to understand how the visual context in which an animal is portrayed influences interest in pet ownership. We showed images of sloths and pythons in naturalistic zoo enclosures, with professional animal care staff, with members of the public, outside of zoo habitats on educational perches, and in typical photo-prop tourism settings. Contrary to our hypotheses, we found no effect of visual context on interest in pet ownership. We recognize that this may be due to the brief, 15-second exposure to the image of interest. In reality, exposure to animals in varying contexts is a recurrent event in one’s life, and degree of exposure depends largely on personal experiences and social and other media practices. This brief exposure may not have been substantial enough to influence perception about the animal in light of respondents’ own histories. Additionally, although past studies have detected differences in interest in pet ownership with brief exposure to images [41, 42], it is also possible that this

specific online survey format in which the stimuli preceded the question did not measurably influence respondents' perceptions in the same way that other study designs have (e.g., those that used physical, in-hand photographs, or those that were available for persistent viewing throughout the survey).

Previous researchers have errantly interpreted a lack of significant effect of context on interest in non-domesticated pet ownership as indication that there is no influence of context [29]. When analyses fail to demonstrate statistical significance, with typical inferential statistics such as used here and in Spooner & Stride, ~~2021~~ [29], it is not statistically nor logically warranted to conclude that two treatments produce the same effect [e.g., 65]. All one can conclude is that the sample did not provide sufficient evidence to conclude that the effect exists. Therefore, unlike Spooner & Stride, ~~2021~~ [29], we do not recommend that our findings be interpreted as evidence that humans in photos do not influence viewers' interest in sloth and python pet ownership. Rather, we acknowledge that additional research and alternative study designs are needed to understand precisely what types of visual experiences engender and discourage interest in non-domesticated pet ownership.

The present study design was intended to measure interest in non-domesticated pet ownership that is associated with viewing other's experiences with animals in educational programs or tourist opportunities. The stimuli in this study are reminiscent of images that would be seen in a social media feed or witnessed as one viewed another person participating in an animal experience. The stimuli do not replicate experiences that people themselves have in an in-person program, such as those experienced at zoos and aquaria. As such, respondents were not exposed

to responsible interpretation or conservation-oriented messaging that characterizes animal programs at many accredited zoos. If and how verbal messaging interacts with visual context effects in zoo-based programs is a topic of current study [recently reviewed in 49, 66, 67], but not one tackled here.

Survey respondents from younger generations reported more agreement that they would like to have a sloth or a python as a pet. With the present cross-sectional (rather than longitudinal) study design, it is not possible to comment on how interest in pet ownership may change with age and whether there are developmental effects influencing the patterns observed here. Given the previous connections identified between media and interest in pet ownership [32-39], one explanation for these findings may be that younger generations characterized by social media use, and regularly exposed to images of these animals in unnatural contexts and alongside humans, are developing more interest in pet ownership than older generations without that long-term exposure. Many people, likely including those participating in our study, are engaged with online platforms and social media regularly. Whether regular exposure to representations of animals in the media explains the persistent interest in non-domesticated pet ownership we report here remains speculation; however, we hope this work fuels future longitudinal research that can appropriately study the causes of interest in non-domesticated pet ownership.

Most previous research has demonstrated increased affinity and more positive attitudes toward animals by females compared to males [55, 68-71]. In general, females tend to place more value on and show more concern for nonhuman species and conservation compared to males, and therefore report more positive wildlife attitudes [68, 72]. Females may also form stronger

emotional connections to individual animals than males [55]. However, for species associated with common phobias and predators, females have reported stronger fears, feelings of disgust, and negative attitudes associated with those particular animals [54, 55, 73, 74]. An affinity for animals and propensity for positive wildlife attitudes may not equate to a desire to have a non-domesticated pet, however, as here we find no gender difference for interest in sloths as pets. We did find the least amount of interest in python ownership among females, which is consistent with stronger phobias reported by females towards predatory species.

The negative influence that non-domesticated pet ownership has on animal welfare and animal conservation is well established. What remains to be determined is how interest in non-domesticated pet ownership develops, and how it can be modulated through decisions that individuals and animal organizations make about both the in-person experiences that they create and the visuals that are associated with these experiences. Designing experiences for people that promote responsible actions and attitudes toward animals and avoid behavioral spillover of generating animal interest that can lead to unintended negative consequences for animals is important as organizations continue to develop ways to connect people to nature [49]. Given the prevalence of interest in non-domesticated pet ownership among the general public measured here, and the persistence of this interest regardless of the visual context, there is a pressing need for additional research with powerful alternative methodologies to better understand the human psychology driving interest in non-domesticated pet ownership.

Conclusion

The trade and private ownership of non-domesticated animals has detrimental effects on individual animals and their wild populations, and this study sought to identify whether brief exposure to common visual portrayals of sloths or pythons contributed to an interest in ownership of these species as pets. Thirty-nine percent of respondents reported interest in sloth pet ownership, 21% reported interest in python pet ownership, and visual context did not significantly affect survey respondents' reported interest in having either species as a pet. However, generation was a significant predictor of interest in both sloth and python pet ownership, with younger generations reporting more interest in having these species as pets. We speculate that the regular media exposure to animals in unnatural contexts that is regularly experienced by younger generations may contribute to the generational effect found here and encourage further research to better understand the ontogeny of interest in non-domesticated pet ownership.

Acknowledgements

We are grateful to Chris Bjalba for creating images, Jen Torchalski for assistance with image design, Lily Maynard and John Andrews for advice on survey design, and S. Sunny Nelson for feedback on the manuscript. We acknowledge the open-source sloth photo created by senivpetro and obtained from www.freepik.com.

References

1. Bush ER, Baker SE, Macdonald DW. Global trade in exotic pets 2006-2012. Conserv Biol. 2014;28(3): 663-76.
2. Scheffers BR, Oliveira BF, Lamb I, Edwards DP. Global wildlife trade across the tree of life. Science. 2019;366(6461): 71-6.
3. Schuppli CA, Fraser D, Bacon HJ. Welfare of non-traditional pets. Rev Sci Tech (International Office of Epizootics). 2014;33(1): 221-31. Epub 2014/07/09.
4. Baker SE, Cain R, Van Kesteren F, Zommers ZA, D'cruze N, Macdonald DW. Rough trade: animal welfare in the global wildlife trade. Bioscience. 2013;63(12): 928-38.
5. Grieser-Johns A, Thomson J. Going, going, gone: the illegal trade in wildlife in East and Southeast Asia. World Bank, Washington, DC. 2005.
6. Haken J. Transnational crime in the developing world. Global financial integrity. 2011;32(2): 11-30.
7. Nijman V. An overview of international wildlife trade from Southeast Asia. Biodivers Conserv. 2010;19(4): 1101-14.
8. Sodhi NS, Koh LP, Brook BW, Ng PKL. Southeast Asian biodiversity: an impending disaster. Trends Ecol Evol. 2004;19(12): 654-60.
9. Carder G, Plese T, Machado FC, Paterson S, Matthews N, McAnea L, et al. The impact of 'selfie'tourism on the behaviour and welfare of brown-throated three-toed sloths. Animals. 2018;8(11): 216.
10. von Essen E, Lindsjš J, Berg C. Instagranimal: animal welfare and animal ethics challenges of animal-based tourism. Animals. 2020;10(10): 1830.

11. Kuhnen V, Kanaan V. Wildlife trade in Brazil: A closer look at wild pets welfare issues. Braz J Biol. 2014;74: 124-7.
12. McMillan SE, Dingle C, Allcock JA, Bonebrake TC. Exotic animal cafes are increasingly home to threatened biodiversity. Conserv Lett. 2021;14(1): e12760.
13. Warwick C, Steedman C, Jessop M, Arena P, Pilny A, Nicholas E. Exotic pet suitability: Understanding some problems and using a labeling system to aid animal welfare, environment, and consumer protection. J Vet Behav. 2018;26: 17-26.
14. Bezerra-Santos MA, Mendoza-Roldan JA, Thompson RA, Dantas-Torres F, Otranto D. Illegal wildlife trade: a gateway to zoonotic infectious diseases. Trends Parasitol. 2021.
15. Can ÖE, D'Cruze N, Macdonald DW. Dealing in deadly pathogens: Taking stock of the legal trade in live wildlife and potential risks to human health. Glob Ecol Conserv. 2019;17: e00515.
16. Jones①]Engel L, Engel GA, Schillaci MA, Froehlich J, Paputungan U, Kyes RC. Prevalence of enteric parasites in pet macaques in Sulawesi, Indonesia. Am J Primatol. 2004;62(2): 71-82.
17. d'Ovidio D, Santoro D. Survey of zoonotic dermatoses in client①]owned exotic pet mammals in southern Italy. Zoonoses Public Health. 2015;62(2): 100-4.
18. Renquist DM, Whitney RA. Zoonoses Acquired from Pet Primates. Vet Clin N Am: Small Anim Pract. 1987;17(1): 219-40.
19. Rosen T, Jablon J. Infectious threats from exotic pets: dermatological implications. Dermatol Clin. 2003;21(2): 229-36. Epub 2003/05/22.
20. Souza MJ. Bacterial and parasitic zoonoses of exotic pets. Vet Clin North Am Exot Anim Pract. 2009;12(3): 401-15, Epub 2009/09/08.

21. Ahmad T, Khan M, Haroon THM, Nasir S, Hui J, Bonilla-Aldana DK, et al. COVID-19: Zoonotic aspects. Travel Med Infect Dis. 2020;36: 101607.

22. Contini C, Di Nuzzo M, Barp N, Bonazza A, De Giorgio R, Tognon M, et al. The novel zoonotic COVID-19 pandemic: An expected global health concern. J Infect Dev Ctries. 2020;14(03): 254-64.

23. Moorhouse TP, Balaskas M, D'Cruze NC, Macdonald DW. Information could reduce consumer demand for exotic pets. Conserv Lett. 2017;10(3): 337-45.

24. Choquette RE, Angulo A, Bishop PJ, Phan C, Rowley J. The internetbased Southeast Asia amphibian pet trade. Traffic Bulletin. 2020;32: 68-76.

25. Kaczmarski M, Kolenda K. Non-native amphibian pet trade via Internet in Poland. Eur J Ecol. 2018;4(1): 30-40.

26. Lavorgna A. Wildlife trafficking in the Internet age. Crime Sci. 2014;3(1): 1-12.

27. Phassaraudomsak M, Krishnasamy K. Trading Faces: a rapid assessment on the use of Facebook to trade in wildlife in Thailand. TRAFFIC, Petaling Jaya, Selangor, Malaysia, 23pp. 2018.

28. Spooner SL, Stride JR. Animal-human two-shot images: Their out-of-context interpretation and the implications for zoo and conservation settings. Zoo Biol. 2021: 1-12.

29. Sung Y-H, Fong JJ. Assessing consumer trends and illegal activity by monitoring the online wildlife trade. Biol Conserv. 2018;227: 219-25.

30. Sy E. Utilisation of Facebook to trade live reptiles in the Philippines. TRAFFIC Report, Petaling Jaya, Selangor, Malaysia, Petaling Jaya, Selangor, Malaysia. 2018.

31. Ghirlanda S, Acerbi A, Herzog H, Serpell JA. Fashion vs. function in cultural evolution: The case of dog breed popularity. PLoS One. 2013;8(9): e74770.

32. Herzog H. Forty-two thousand and one Dalmatians: Fads, social contagion, and dog breed popularity. Soc Anim. 2006;14(4): 383-97.

33. Kitade T, Naruse Y. Otter Alert: A rapid assessment of illegal trade and booming demand in Japan. TRAFFIC Japan Office. 2018.

34. Liska J. Communicating nature: Wild animals in the living room. Anthrozošs. 1999;12(2): 88-96.

35. Mitchell M, Tully TN. Manual of Exotic Pet Practice-E-Book. Elsevier Health Sciences; 2008 Mar 4.

36. Moloney GK, Tuke J, Dal Grande E, Nielsen T, Chaber A-L. Is YouTube promoting the exotic pet trade? Analysis of the global public perception of popular YouTube videos featuring threatened exotic animals. PLoS One. 2021;16(4): e0235451. doi: 10.1371/journal.pone.0235451.

37. Nekaris BKA-I, Campbell N, Coggins TG, Rode EJ, Nijman V. Tickled to death: analysing public perceptions of 'cute'videos of threatened species (slow lorises-Nycticebus spp.) on Web 2.0 Sites. PLoS One. 2013;8(7): e69215.

38. Redmalm D. Holy bonsai wolves: Chihuahuas and the Paris Hilton syndrome. Int J Cult Stud. 2014;17(1): 93-109.

39. Siriwat P, Nijman V. Illegal pet trade on social media as an emerging impediment to the conservation of Asian otters species. J Asia Pac Biodivers. 2018;11(4): 469-75.

40. Schroepfer KK, Rosati AG, Chartrand T, Hare B. Use of ÒEntertainmentÓ Chimpanzees in Commercials Distorts Public Perception Regarding Their Conservation Status. PLoS One. 2011;6(10): e26048.

41. Ross SR, Vreeman VM, Lonsdorf EV. Specific Image Characteristics Influence Attitudes about Chimpanzee Conservation and Use as Pets. PLoS One. 2011;6(7): e22050.

42. Leighty KA, Valuska AJ, Grand AP, Bettinger TL, Mellen JD, Ross SR, et al. Impact of visual context on public perceptions of non-human primate performers. PLoS One. 2015;10(2): e0118487.

43. Binding S. Picture this. Zooquaria Winter 2018/19. 2018:12.

44. International Primatological Society. 2021 [cited 2021 June 25]. Available from: http://www.internationalprimatologicalsociety.org/OppositionToTheUseOfNonhumanPrimatesInTheMedia.cfm.

45. Waters S, Setchell JM, MarŽchal L, Oram F, Cheyne SM. The IUCN Primate Specialist Group Section for Human-Primate Interactions Best Practice Guidelines for Responsible Images of Non-Human Primates. Ouwehand Zoo Foundation, The Netherlands.

46. D'Cruze N, Paterson S, Green J, Megson D, Warwick C, Coulthard E, et al. Dropping the ball? The welfare of ball pythons traded in the EU and North America. Animals. 2020;10(3): 413.

47. Akerman S. Best practices for building empathy through live animal encounters. J Mus Educ. 2019;44(1): 89-95.

48. Godinez AM, Fernandez EJ. What is the zoo experience? How zoos impact a visitor's behaviors, perceptions, and conservation efforts. Front Psychol. 2019;10: 1746.

49. Learmonth MJ. Human-Animal Interactions in Zoos: What Can Compassionate Conservation, Conservation Welfare and Duty of Care Tell Us about the Ethics of Interacting, and Avoiding Unintended Consequences? Animals. 2020;10(11): 2037.

50. Janssen J, Gomez L. An examination of the import of live reptiles from Indonesia by the United States from 2000 to 2015. J Nat Conserv. 2021;59: 125949.
51. Moreno S, Plese T. The illegal traffic in sloths and threats to their survival in Colombia. Edentata. 2006;7: 10-18.
52. Green J, Coulthard E, Megson D, Norrey J, Norrey L, Rowntree JK, et al. Blind trading: A literature review of research addressing the welfare of Ball pythons in the exotic pet trade. Animals. 2020;10(2): 193.
53. Serpell JA. Factors influencing human attitudes to animals and their welfare. Anim Welfare. 2004;13(1): 145-51.
54. Kellert SR. American attitudes toward and knowledge of animals: An update. Advances in animal welfare science 1984: Springer; 1985. p. 177-213.
55. Kellert SR, Berry JK. Attitudes, knowledge, and behaviors toward wildlife as affected by gender. Wildlife Society Bulletin (1973-2006). 1987;15(3): 363-71.
56. Taylor N, Signal T. Attitudes to Animals in the Animal Protection Community Compared to a Normative Community Sample. Soc Anim. 2006;14(3): 265-74.
57. Signal T, Taylor N, Maclean AS. Pampered or pariah: does animal type influence the interaction between animal attitude and empathy? Psychol Crime Law. 2018;24(5): 527-37.
58. Mason W, Suri S. Conducting behavioral research on Amazon's Mechanical Turk. Behav Res Methods. 2012;44(1): 1-23.
59. R Core Team. R: a language and environment for statistical computing. R Foundation for Statistical Computing, Vienna, Austria. https://www. R-project. org. Vienna, Austria. 2020.
60. Venables W, Ripley B. BD (2002). Modern Applied Statistics with S. New York: Springer Science & Business Media. 2002;200: 183-206.

61. Dimock M. Defining generations: Where Millennials end and Generation Z begins. Pew Research Center. 2019;17(1): 1-7.
62. Brant R. Assessing proportionality in the proportional odds model for ordinal logistic regression. Biometrics. 1990: 1171-8.
63. Fox J, Hong J. Effect displays in R for multinomial and proportional-odds logit models: Extensions to the effects package. J Stat Softw. 2009;32(1): 1-24.
64. Fox J, Weisberg S. An R companion to applied regression: Sage publications; 2018.
65. Greenland S, Senn SJ, Rothman KJ, Carlin JB, Poole C, Goodman SN, et al. Statistical tests, P values, confidence intervals, and power: a guide to misinterpretations. Eur J Epidemiol. 2016;31(4): 337-50.
66. de Mori B, Ferrante L, Florio D, Macchi E, Pollastri I, Normando S. A protocol for the ethical assessment of wild animal-visitor interactions (AVIP) evaluating animal welfare, education, and conservation outcomes. Animals. 2019;9(8): 487.
67. Spooner SL, Farnworth MJ, Ward SJ, Whitehouse-Tedd KM. Conservation Education: Are zoo animals effective ambassadors and is there any cost to their welfare? J Zool Bot Gard. 2021;2(1): 41-65.
68. Czech B, Devers PK, Krausman PR. The relationship of gender to species conservation attitudes. Wildl Soc Bull. 2001: 187-94.
69. Herzog HA. Gender differences in human-animal interactions: A review. Anthrozošs. 2007;20(1): 7-21.
70. Naylor W, Parsons ECM. An online survey of public knowledge, attitudes, and perceptions toward whales and dolphins, and their conservation. Front Mar Sci. 2018;5: 153.

71. Mathews S, Herzog HA. Personality and attitudes toward the treatment of animals. Soc Anim. 1997;5(2): 169-75.

72. Leuschner WA, Ritchie VP, Stauffer DF. Opinions on wildlife: responses of resource managers and wildlife users in the Southeastern United States. Wildl Soc Bull. (1973-2006). 1989;17(1): 24-9.

73. Fredrikson M, Annas P, Fischer H, Wik G. Gender and age differences in the prevalence of specific fears and phobias. Behav Res Ther. 1996;34(1): 33-9.

74. Myers Jr OE, Saunders CD, Birjulin AA. Emotional dimensions of watching zoo animals: An experience sampling study building on insights from psychology. Curator (N Y). 2004;47(3): 299-321.

75. Lindqvist A, SendŽn MG, Renstršm EA. What is gender, anyway: a review of the options for operationalising gender. Psychol Sex. 2020: 1-13.

Supporting Information

S1 Table. Self-reported Genders. This table provides participants' responses to the question "Which of the following best describes your gender?" Response options were "female," "male," "other gender," "prefer to self-describe," and "prefer not to say." In the case that respondents selected "prefer to self-describe," an open text field for self-description was offered. For statistical analysis, genders for analysis were "male," "female," and "other" following [75].

S1 Text. Additional Context Evaluation for Sloth. This text provides an overview of an additional visual context that was evaluated by survey respondents to inform internal decision-making at Lincoln Park Zoo.

S1 Figure. Visual Stimulus Extracted from the Lincoln Park Zoo program "Lettuce with Luigi."

S2 Table. Likelihood Ratio Test Results for Ordinal Logistic Regression Model Including the "LWL" Condition. The results of the likelihood ratio test on the data set that includes the LWL condition are similar to the results obtained without the LWL condition (reported in the main text). Again, there is a significant effect of generation, and context and gender are not statistically significant.

S3 Table. Ordinal Logistical Regression Results for the Sloth Experiment Including the "LWL" condition. The reference value for visual context was the control condition, the reference value for gender was female, and the reference value for generation was Gen Z. Original coefficients are scaled in terms of logs and we provide the exponentiated odds ratios as well.

S2 Figure. Predictor Effects Plot Showing the Role of Visual Context on Interest in Sloth Ownership. Predictor effect plots provide graphical summaries for fitted regression models by averaging and conditioning the other predictor variables to summarize the role of a selected focal predictor in a fitted regression model [64].

S1 Dataset. SlothData_Deposit. This csv file contains the sloth survey data analyzed in the main text.

S2 Dataset. PythonData_Deposit. This csv file contains the python survey data analyzed in the main text.

S3 Dataset_SlothDataLWL_Deposit. This csv file contains the additional sloth data for the "Lettuce With Luigi" condition analyzed in the Supporting Information.

Tables

Table 1. Proportion of Responses by Level of Agreement with the Statement, "I would like to have a sloth/python as a pet."

	Strongly Agree	Agree	Disagree	Strongly Disagree
Sloth	0.112	0.276	0.353	0.260
Python	0.044	0.165	0.245	0.546

Table 1 Legend. Proportions of response selections per species, collapsing across generations and genders.

Table 2. Likelihood Ratio Test Results for Ordinal Logistic Regression Models.

**Sloth Model**	χ^2	**df**	**P-value**
Fixed Factors			
Context	3.872	5	0.5680
Gender	4.459	2	0.1076
**Generation**	**59.676**	**5**	**1.418 e-11**
**Python Model**	χ^2	**df**	**P-value**
Fixed Factors			
Context	5.897	5	0.3163
**Gender**	**24.441**	**2**	**4.929 e-06**
**Generation**	**63.938**	**5**	**1.861 e-12**

Table 2 Legend. Results of the likelihood ratio tests isolating the contributions of the fixed effects in both the sloth and python models are shown in the table. The factors in bold are significant predictors of interest in pet ownership.

Table 3. Ordinal Logistic Regression Results for the Sloth Experiment.

Predictor	Coefficient	Lower-95	Upper-95	S.E.	Odds Ratio
Context_Naturalistic Zoo Habitat	-0.0323	-0.3280	0.2633	0.1509	0.9682
Context_Keeper Contact	-0.1580	-0.4526	0.1363	0.1502	0.8538
Context_Educational Perch	0.0639	-0.2317	0.3595	0.1508	1.0660
Context_Educational Perch with Visitor Contact	0.1100	-0.1854	0.4055	0.1507	1.1163
Context_Public Setting (Yoga)	-0.0346	-0.3314	0.2621	0.1514	0.9660
Gender_Male	0.0119	-0.1595	0.1833	0.0875	1.0120
Gender_Other	-1.0316	-2.035	-0.0639	0.4962	0.3565
Generation_ Millennial	-0.0298	-0.3454	0.2857	0.1609	0.9707
Generation_ Gen X	-0.3772	-0.7194	-0.0355	0.1744	0.6858
Generation_Boomers II	-0.9426	-1.3571	-0.5305	0.2108	0.3896
Generation_Boomers I	-1.3510	-1.9704	-0.7457	0.3116	0.2590
Generation_Post War	-1.3556	-3.0201	0.1433	0.7812	0.2578

Table 3 Legend. The reference value for visual context was the control condition, the reference value for gender was female, and the reference value for generation was Gen Z. Original coefficients are scaled in terms of logs and we provide the exponentiated odds ratios as well.

Table 4. Ordinal Logistic Regression Results for the Python Experiment.

**Predictor**	**Coefficient**	**Lower-95**	**Upper-95**	**S.E.**	**Odds Ratio**
Context_Naturalistic Zoo Habitat	-0.1501	-0.4694	0.1691	0.1629	0.8606
Context_Keeper Contact	-0.0696	-0.3870	0.2477	0.1619	0.9327
Context_Educational Perch	0.1709	-0.1359	0.4776	0.1565	1.1863
Context_Educational Perch with Visitor Contact	0.0059	-0.3061	0.3179	0.1592	1.0060
Context_Public Setting (Tourist Photo)	0.1555	-0.1548	0.4658	0.1583	1.1682
Gender_Male	0.4722	0.2882	0.6562	0.0939	1.6035
Gender_Other	1.0356	0.2167	1.8545	0.4178	2.8168
Generation_ Millennial	0.0168	-0.2882	0.3219	0.1556	1.0170
Generation_ Gen X	-0.1529	-0.4916	0.1858	0.1728	0.8582
Generation_Boomers II	-0.9140	-1.3586	-0.4693	0.2269	0.4009
Generation_Boomers I	-1.6722	-2.4033	-0.9411	0.3730	0.1878
Generation_Post War	-13.1193	-495.2670	469.0285	245.9937	0.000

Table 4 Legend. The reference value for visual context was the control condition, the reference value for gender was female, and the reference value for generation was Gen Z. Original coefficients are scaled in terms of logs and we provide the exponentiated odds ratios as well.

Figure 1. Experimental Conditions, Visual Stimuli, and Sample Size Per Condition. Conditions presented in the online survey in a between-participants design, and number of subjects in each condition. An additional condition was run for internal evaluation of a Lincoln Park Zoo program; details are in Supporting Information.

Figure 2 A-C. Predictor Effect Plots Showing the Role of Each Predictor on Interest in Sloth Ownership. Predictor effect plots provide graphical summaries for fitted regression models by averaging and conditioning the other predictor variables to summarize the role of a selected focal predictor in a fitted regression model (Fox & Weisberg, 2019). (Note: figures sized to span two columns)

Figure 3 A-C. Predictor Effect Plots Showing the Role of Each Predictor on Interest in Python Ownership. Predictor effect plots provide graphical summaries for fitted regression models by averaging and conditioning the other predictor variables to summarize the role of a selected focal predictor in a fitted regression model (Fox & Weisberg, 2019). (Note: figures sized to span two columns)

Click here to access/download;Figure;Fig 1. Conditions & Stimuli.tif

Condition & Label	Visual Context	Sloth Stimulus & Sample Size	Python Stimulus & Sample Size
Control<br>"Control"	Animal without any visual context	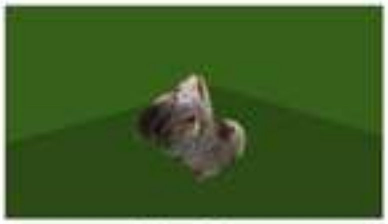<br>N = 293	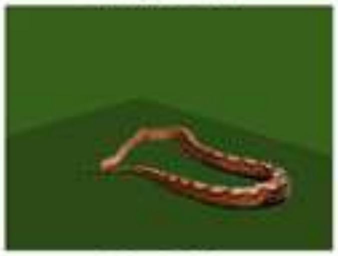<br>N = 295
Naturalistic Zoo Habitat<br>"Habitat"	Animal residing in a naturalistic zoo habitat	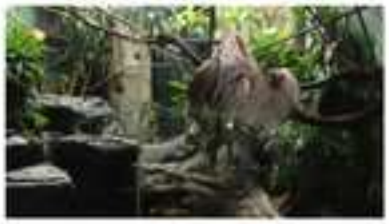<br>N = 291	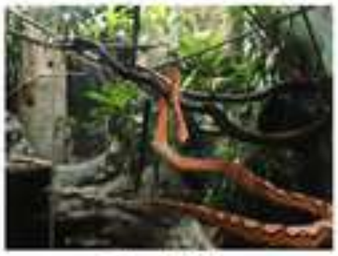<br>N = 295
Zoo Keeper Contact<br>"Zoo Keeper"	Animal presented in contact with a uniformed zoo keeper	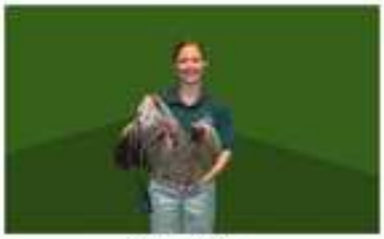<br>N = 296	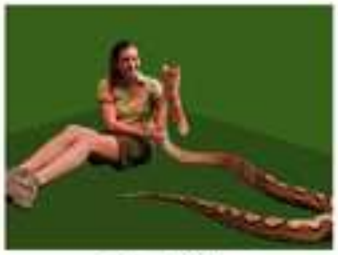<br>N = 294
Educational Perch<br>"Perch"	Animal on an educational perch	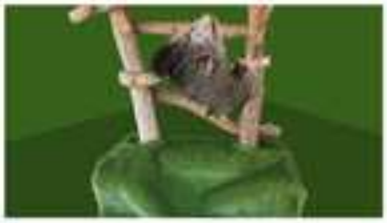<br>N = 294	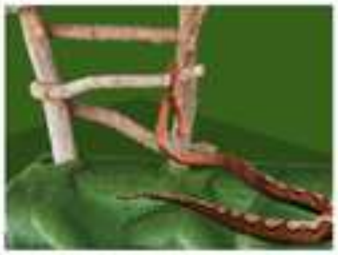<br>N = 301
Educational Perch with Visitor Contact<br>"Touch"	Animal on an educational perch being touched by a member of the public	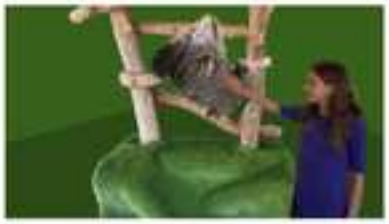<br>N = 299	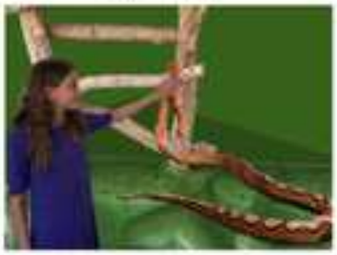<br>N = 302
Public Setting<br>"Yoga" or "Tourist Photo"	Animal in visual context of "sloth yoga" or a tourist photo with a python	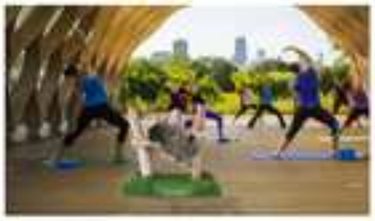<br>N = 294	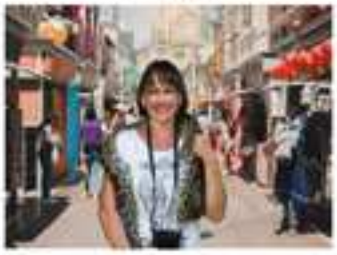<br>N = 298

Click here to access/download;Figure;Fig 2. Predicted Probability Plots Sloths.tif

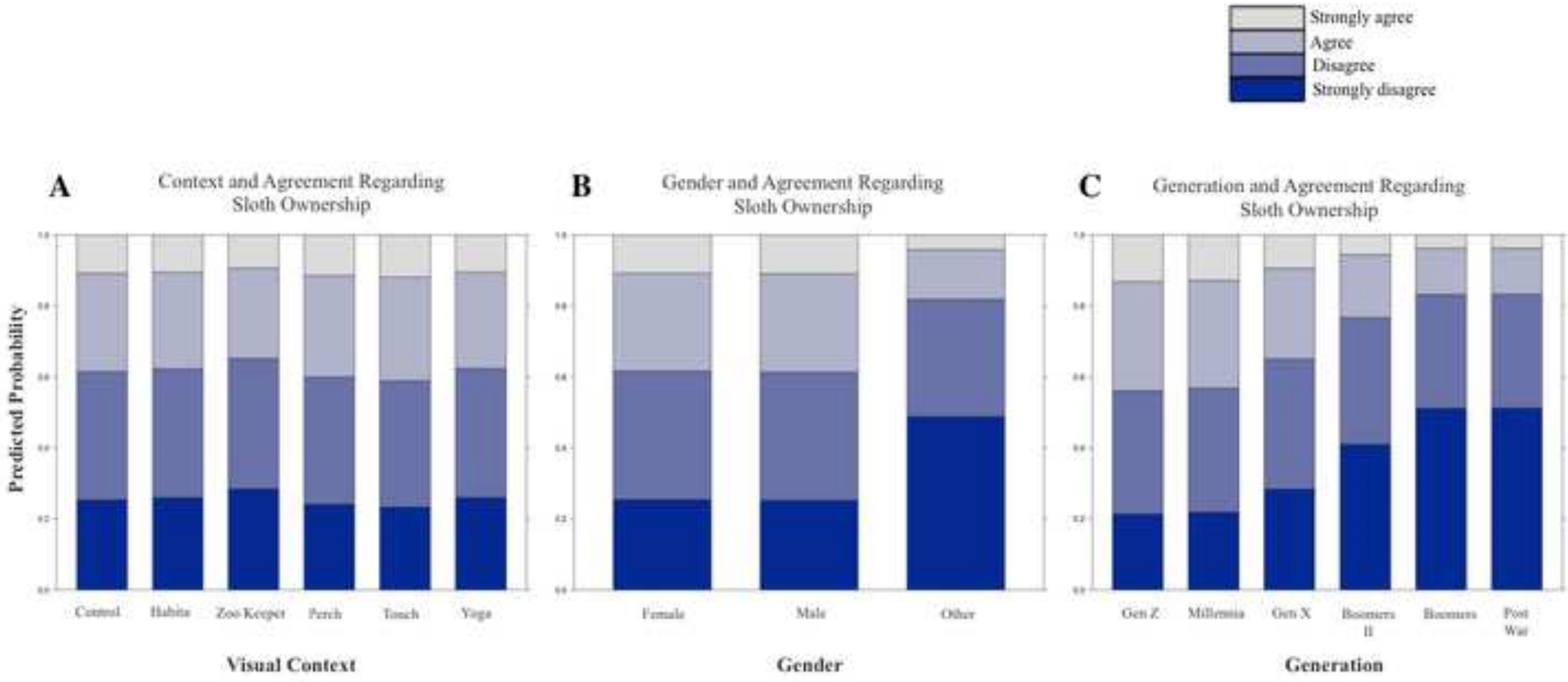

Click here to access/download;Figure;Fig 3. Predicted Probability Plots Pythons.tif

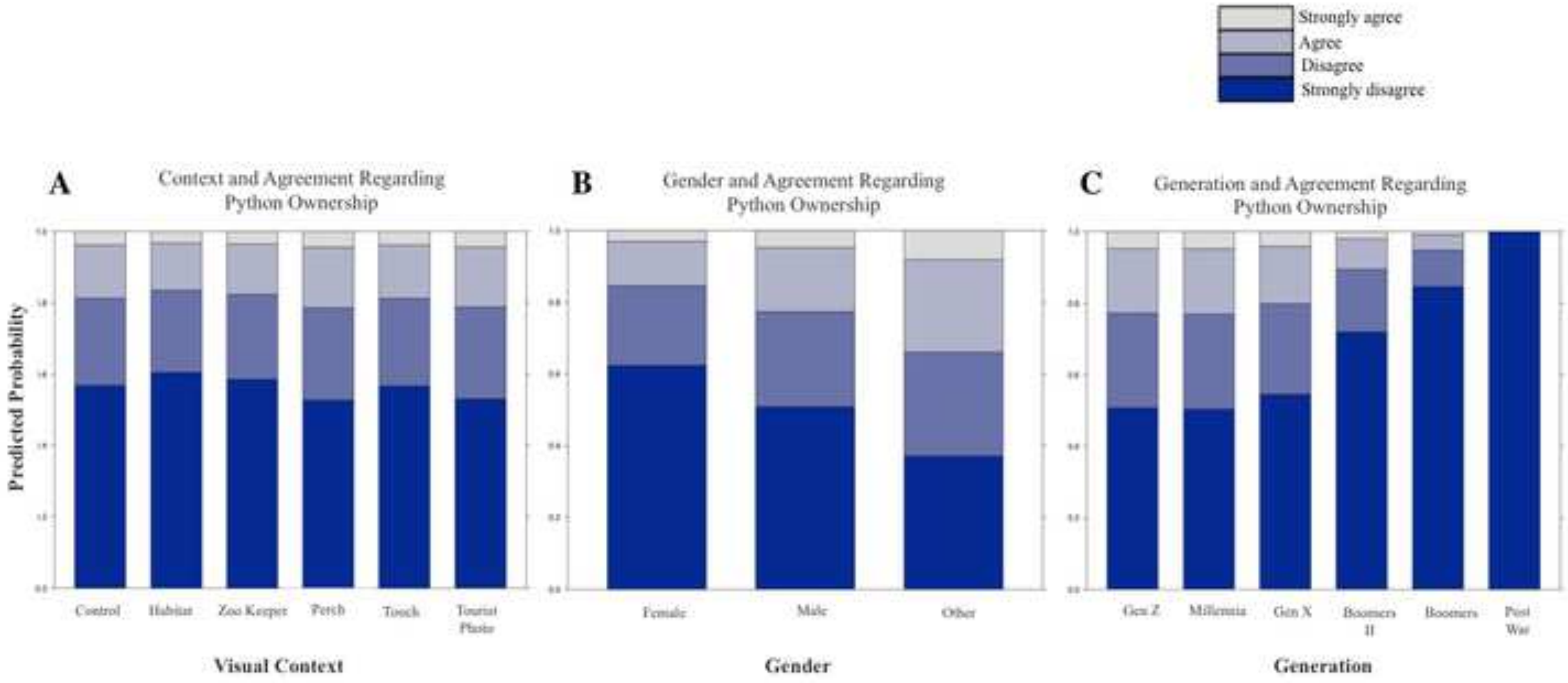

Click here to access/download
Supporting Information
Portrayals SI_30Aug21.pdf

Click here to access/download
Supporting Information
SlothData_Deposit.csv

Click here to access/download
Supporting Information
PythonData_Deposit.csv

Click here to access/download
Supporting Information
SlothDataSI_Deposit.csv
